# Expression of muscarinic receptor subtypes in tree shrew ocular tissues and their regulation during the development of myopia

**Published:** 2009-03-02

**Authors:** N.A. McBrien, A.I. Jobling, H.T. Truong, C.L. Cottriall, A. Gentle

**Affiliations:** Department of Optometry and Vision Sciences, the University of Melbourne, Victoria, Australia

## Abstract

**Purpose:**

Muscarinic receptors are known to regulate several important physiologic processes in the eye. Antagonists to these receptors such as atropine and pirenzepine are effective at stopping the excessive ocular growth that results in myopia. However, their site of action is unknown. This study details ocular muscarinic subtype expression within a well documented model of eye growth and investigates their expression during early stages of myopia induction.

**Methods:**

Total RNA was isolated from tree shrew corneal, iris/ciliary body, retinal, choroidal, and scleral tissue samples and was reverse transcribed. Using tree shrew-specific primers to the five muscarinic acetylcholine receptor subtypes (*CHRM1*-*CHRM5*), products were amplified using polymerase chain reaction (PCR) and their identity confirmed using automated sequencing. The expression of the receptor proteins (M1-M5) were also explored in the retina, choroid, and sclera using immunohistochemistry. Myopia was induced in the tree shrew for one or five days using monocular deprivation of pattern vision, and the expression of the receptor subtypes was assessed in the retina, choroid, and sclera using real-time PCR.

**Results:**

All five muscarinic receptor subtypes were expressed in the iris/ciliary body, retina, choroid, and sclera while gene products corresponding to *CHRM1*, *CHRM3*, *CHRM4*, and *CHRM5* were present in the corneal samples. The gene expression data were confirmed by immunohistochemistry with the M1-M5 proteins detected in the retina, choroid, and sclera. After one or five days of myopia development, muscarinic receptor gene expression remained unaltered in the retinal, choroidal, and scleral tissue samples.

**Conclusions:**

This study provides a comprehensive profile of muscarinic receptor gene and protein expression in tree shrew ocular tissues with all receptor subtypes found in tissues implicated in the control of eye growth. Despite the efficacy of muscarinic antagonists at inhibiting myopia development, the genes of the muscarinic receptor subtypes are neither regulated early in myopia (before measurable axial elongation) nor after significant structural change.

## Introduction

Muscarinic acetylcholine receptors (mAChRs) are a group of neurotransmitter proteins belonging to the seven transmembrane superfamily of receptors. Five distinct receptor genes (*CHRM1*-*CHRM5*) have been cloned, which encode for five separate muscarinic receptor proteins (M1-M5), each with specific pharmacological properties [1-3]. Numerous studies have detailed a wide distribution for these receptors throughout the central and peripheral nervous systems. However, there are tissue-specific subtype expression patterns [4-6]. Studies have shown mAChRs are responsible for diverse physiologic responses such as increased salivation, smooth muscle contraction, memory, and cardiac function [7-10] while drugs targeting the muscarinic receptors have been developed for the treatment of pathological conditions such as Alzheimer disease, asthma, disorders of intestinal motility, and cardiac dysfunction [2].

Within the eye, mAChR genes and proteins have been identified in several tissues and play critical roles in ocular responses [11,12]. The ciliary body and iris sphincter express all five receptor subtypes with the M3 subtype being predominant [13-15]. Parasympathetic stimulation of these tissues result in accommodation, altered aqueous outflow, and contraction of the iris sphincter muscle [16,17]. While corneal muscarinic receptors have been shown to be involved in regulating growth of the corneal epithelium and have been proposed to control corneal wound healing [18], innervation of the choroid leads to blood vessel dilation, principally through the M3 and possibly M5 receptor subtypes [19]. Muscarinic receptors are also thought to be involved in regulating retinal embryonic and postnatal development [20-23] as well as modulating the activity of directionally-sensitive ganglion cells [24,25].

In addition to the functional effects of mAChRs described above, these receptors may also be involved in the postnatal control of ocular growth. Numerous studies across a range of animal models of myopia development have demonstrated that the non-selective antagonist, atropine, is effective in preventing the axial elongation that leads to myopia development [26-29]. Human clinical trials have also detailed the effectiveness of daily atropine administration, reducing the progression of myopia by approximately 60% at least in the first year of treatment [30,31]. Another non-selective muscarinic antagonist, oxyphenonium, has also been reported to inhibit myopia development in chicks [32]. In an attempt to reduce side effects and provide information on the mechanism of action, more specific muscarinic antagonists have been investigated for their effectiveness at reducing myopia. Investigations have revealed only the M1 selective pirenzepine and M2/M4 selective himbacine inhibiting myopia development [29,33,34].

Despite the effectiveness of muscarinic antagonists at inhibiting myopia, their mechanism of action remains unclear. While studies involving selective antagonists suggest an involvement of the M1 and/or M4 receptor, the high drug concentrations required to prevent myopia may suggest involvement of a non-cholinergic receptor mechanism such as the nicotinic system [33-35]. This uncertainty is compounded by the limited information on the exact site of action of these drugs. While experimental evidence suggests ocular growth is regulated locally [36], implicating the retina, choroid, and/or sclera, the role of the retinal acetylcholine system in eye growth has been discounted by Fischer et al. [37]

While the mAChR subtype expression has been investigated in the cornea, iris, ciliary body, and retina using pharmacological, protein, and molecular techniques [11,15,18,22],  there is relatively less information on choroidal and scleral expression. This is particularly the case in mammalian models of myopia development where the characterization of expression may aid in elucidating their effect on eye growth. While relatively recent evidence highlights the expression of the five receptor subtypes in the sclera [38], radioligand binding experiments report no change in muscarinic receptor density after two and seven days of myopia induction in the chick [39]. This is not supported by mRNA data reporting a selective *mAChR* subtype regulation in the sclera at a later stage in myopia development (after 21 days) in the guinea pig [40]. Such data may highlight species specific differences in the mAChR system or reflect a downstream regulation of the receptors due to increased eye size. If the mAChRs are involved in the development of myopia as suggested by the capacity of antagonists to inhibit axial elongation in humans, a characterization of the temporal nature of mAChR expression during the early stages of myopia is required. Furthermore, examination of the mAChRs in different models of ocular growth may highlight species-specific differences.

The present study details the expression profile of the five mAChR subtypes in tree shrew ocular tissues using reverse transcription polymerase chain reaction (RT–PCR) and immunohistochemistry. The tree shrew is a well documented model in the understanding of eye growth and has been shown to exhibit numerous similarities to human [41]. As mAChRs may be involved in the control of eye growth and the regulation of these receptors during myopia development is equivocal, the gene expression of the five subtypes was assessed in the retina, choroid, and sclera at early time points before and after measurable changes in axial length. Since these ocular tissues are thought to be involved in the control of eye growth, this study provides further insight into the role of muscarinic receptors and their antagonists in the control of myopia development.

## Methods

### Experimental model and paradigm

Maternally reared tree shrews (*Tupaia belangeri*) were used in this study since they have similar ocular structure to humans and are a well documented mammalian model for myopia [41,42]. Animals were used 15 days after natural eye opening, a time when they are particularly susceptible to eye growth changes [41]. Myopia was induced monocularly in two groups of animals for one or five days, using a translucent occluder attached to a head mounted goggle (n=6 for each group). A third group of animals were age matched (20 days post eye opening, n=6) and served as a non-visually manipulated (binocularly normal) control group. Lighting was on a 14:10 h light:dark cycle, illumination was approximately 250 lux at cage floor level, and food and water were available ad libitum. Retinoscopy and A-scan ultrasonography measures were taken under anesthesia at baseline and after the specific treatment durations previously described [43]. All experimental protocols conformed to the ARVO statement for the Use of Animals in Ophthalmic and Vision Research.

### Ocular tissue collection

Animals were deeply anesthetized with a mixture of ketamine (90 mg/kg) and xylazine (10 mg/kg), ocular measures were taken, and a terminal dose of sodium pentobarbital (120 mg/kg) was administered. The eyes were enucleated and hemisected, and the iris/ciliary body complex (iris/ciliary body) and a 5 mm central corneal button were collected. The posterior eyecup was flat mounted, and a surgical trephine was used to isolate a 7 mm section from which the retina, choroid, and posterior sclera were isolated. To prevent contamination of the sclera with neural tissue, the optic nerve head was removed while the neural retina contained minimal retinal pigment epithelium (<20% of total epithelium) after dissection. The remaining epithelial cells were removed from choroidal samples using filter paper. All dissected tissue samples were immediately frozen in liquid nitrogen and stored at −80 °C until use.

### Scleral fibroblast cell culture

Total sclera was isolated and placed in a culture vessel (Nunc, Roskilde, Denmark) containing Dulbecco’s modification of Eagle’s medium (DMEM; Invitrogen, Carlsbad, CA) supplemented with 10% fetal calf serum (FCS), 25 mM HEPES, 100 U/ml penicillin and 100 μg/ml streptomycin (JRH, Melbourne, Australia). Cell outgrowth from explants was observed after approximately one week, and confluence was generally reached after 2.5 weeks.

### RNA extraction and reverse transcription

Total RNA was extracted from the tissue samples using the phenol-chloroform extraction method of Chomczynski et al. [44]. To limit genomic contamination, samples were treated with DNase 1 (Promega Corp., Madison WI) before undergoing a second phenol-chloroform extraction. Commercial spin columns (RNeasy; Qiagen, Valencia, CA) were used to isolate total RNA from the scleral fibroblast samples according to the manufacturer’s instructions. The concentration and purity of total RNA was assessed using an ultraviolet (UV) spectrophotometer (Shimadzu, Kyoto, Japan). Samples (0.5 μg) were reverse transcribed using a commercial reverse transcriptase and 1 μM oligo dT_15_ primer (M-MLV; Promega Corp.).

### Muscarinic receptor subtype gene expression

Fragments of the five *mAChR* genes were amplified using PCR and incorporating tree shrew-specific primers (Table 1). These primers were designed using partial tree shrew muscarinic receptor sequences obtained by direct sequencing of amplification products that were generated using human-specific primers [45]. At least one of the oligonucleotides in each subtype primer pair was designed to the variable cytoplasmic loop 3 in the respective subtypes [46]. Amplification (PCR Express; Hybaid, Ashford, UK) of the muscarinic receptor products was achieved using a commercial ‘hotstart’ polymerase (HotStarTaq master mix; Qiagen, Valencia, CA) and 1 μM of the respective primers. For the majority of samples, 1.5mM MgCl_2_ was used in the reaction, but different concentrations were required for retinal *CHRM3* (2 mM), corneal *CHRM1* (3 mM), and retinal *CHRM5* (3 mM) amplifications to optimise product amplification. After an initial denaturation at 95 °C for 10 min, the samples underwent 40 cycles of denaturation (95 °C, 30 s), annealing (55 °C, 30 s), and extension (72 °C, 1 min). This was followed by a final extension step at 72 °C for 10 min. Individual products were subsequently purified (Qiaquick; Qiagen) and sequenced (CEQ 8000 Genetic Analysis System; Beckman Coulter, Fullerton, CA) to confirm identity.

**Table 1:** Muscarinic receptor subtype oligonucleotide primers and optimized real-time PCR conditions.

**Receptor subtype**	**Oligonucleotide primers (5′-3′)**		**Size (bp)**	**Real-time PCR conditions**		
	**Forward**	**Reverse**		**MgCl_2_ (mM)**	**Ann temp (°C)**	**Det temp (°C)**
*CHRM1*	GGAAGAGGAGAGGGATGAAGG	TTCGGGAACACAGTCCTTG	365	2	63	89
*CHRM2*	TCATGACTGCGCTCTATTGG	GGGCTTTTCCATTGTGGAT	197	3	60	82
*CHRM3*	GCCATCTTGTTCTGGCAATAC	AGCGGCCATACTTCCTCC	325	2	60	82
*CHRM4*	ACACCGTGTACATCGTCAAG	CAGGAGATGTGGATGTAGAGC	394	2	60	86
*CHRM5*	CCAACTCACAGGGCTTTTCT	GTGAGCTGCTCGGCTTTG	173	3	60	87

Tree shrew-specific primers were designed for the five *mAChR* subtypes and were used to detect mRNA expression in the cornea, iris/ciliary body, retina, choroid, sclera, and scleral fibroblasts. The optimized conditions used to assess gene expression changes during myopia development are also included. Under “Real-time PCR conditions”, “Ann temp” stands for annealing temperature and “Det temp” stands for signal detection temperature.

### Muscarinic receptor immunohistochemistry

For detection of a mAChR protein, flat mounted posterior eyecups from age-matched (20 days post eye opening, n=4) binocularly normal tree shrews were fixed in 4% paraformaldehyde in phosphate buffer (0.1 M, pH 7.4) at room temperature for 30 min. Samples were cryoprotected in ascending concentrations of sucrose (10%, 20%, and 30% in phosphate buffer) each for 1 h and then incubated overnight in 30% sucrose. The tissue sections containing retina, choroid, and sclera were embedded (OCT compound, Tissue-Tek; Sakura Finetek, Torrance, CA), and 12 µm sections were cut at −20 °C (Leica CM3050 S; Leica, Heidelberg, Germany). Multiple sections incorporating the posterior pole were collected on poly-L-lysine coated slides (Menzel-Glazer, Braunschweig, Germany) and stored at −20 °C until use.

Commercial immunofluorescent assay kits (IFA muscarinic receptor kits; Research & Diagnostic Antibodies, Benicia, CA) were used according to the manufacturer’s instructions to detect muscarinic receptor protein expression in the retina, choroid, and sclera. Controls were incubated with primary antibodies that had been blocked with the appropriate neutralizing antigen (Research & Diagnostic Antibodies). Sections were observed microscopically (Axioplan/Axiophot 2; Zeiss, Oberkochen, Germany) using a 450–490 nm filter.

### Real-time polymerase chain reaction

To quantify gene expression changes during myopia development, real-time PCR (LightCycler; Roche, Mannheim, Germany) was used. Total RNA (0.5 μg) was reverse transcribed as described above, and amplifications were performed using a commercial PCR mix incorporating SYBR green 1 (FastStart DNA Master Mix; Roche). The protocol for each receptor subtype was optimized to ensure that a single, specific product was produced (Table 1). The melting temperatures for *CHRM1* (92 °C), *CHRM2* (86 °C), *CHRM3* (88 °C), *CHRM4* (92 °C), and *CHRM5* (91 °C) PCR products were empirically determined. Changes in gene expression were assessed with reference to the housekeeping gene, hypoxanthine phosphoribosyl transferase (*HPRT*), as described previously [47]. Group mean data were expressed as the percentage difference between treated and control eyes (±SEM) for the myopia-induced group or left and right eyes (±SEM) for the normal group. Data were analyzed using GraphPad Prism software (GraphPad Software Inc., La Jolla, CA).

### Statistics

The retinoscopy and A-scan ocular data were assessed using *t*-tests and one-way analysis of variance (ANOVA) for within and between treatment groups, respectively. For the gene expression data, *t*-tests were used to assess treated–control differences within each group while the normal, 1 day and 5 day groups were compared using one way ANOVA.

## Results

**Figure 1:**
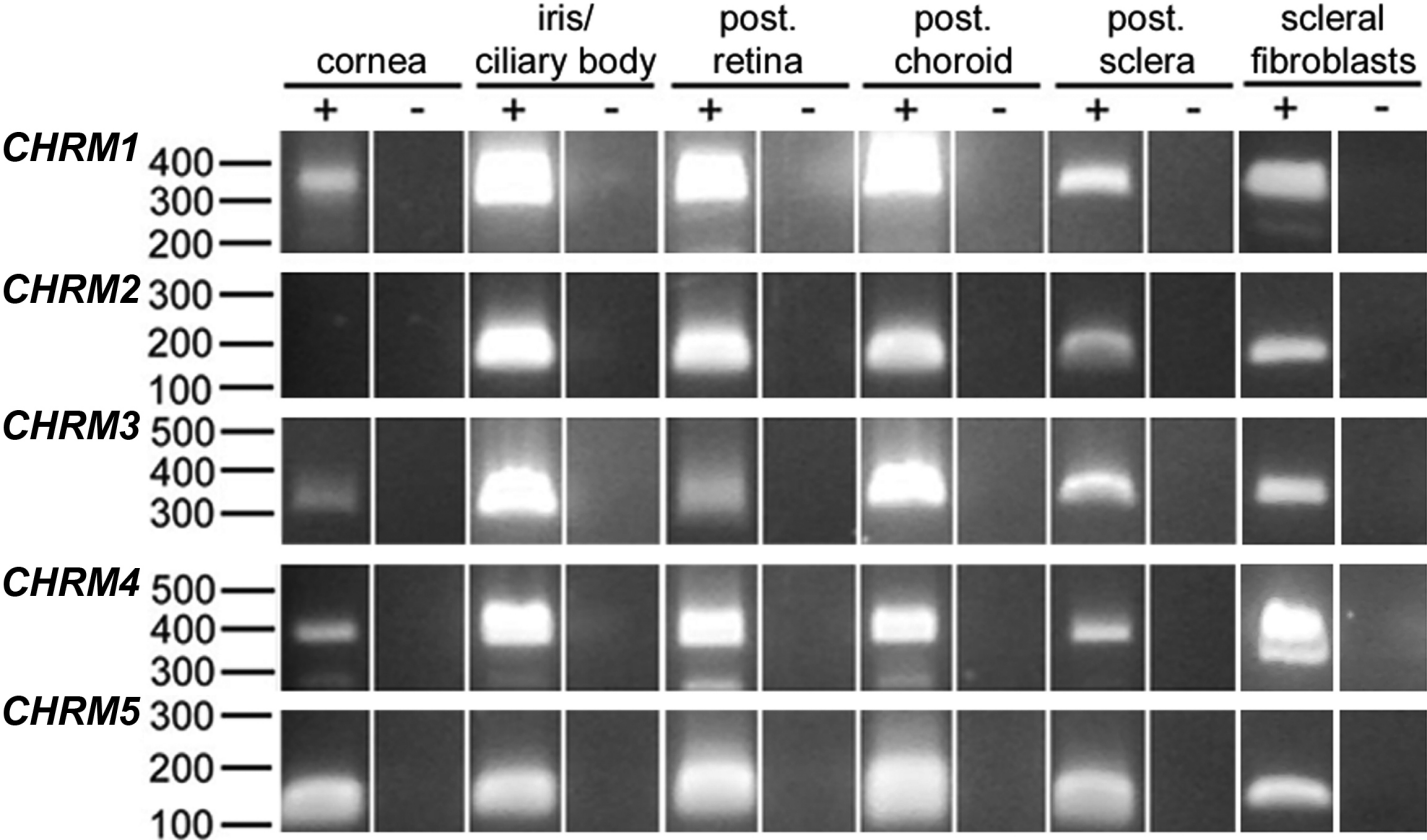
Muscarinic receptor subtype gene expression in the tree shrew eye. RT–PCR and tree shrew-specific primers were used to amplify *mAChR* subtypes in tree shrew corneal, iris/ciliary body, retinal, choroidal, and scleral tissue samples as well as in cultured scleral fibroblasts. Products corresponding to the muscarinic receptor subtypes, *CHRM1* (365 bp), *CHRM2* (197 bp), *CHRM3* (325 bp), *CHRM4* (394 bp), and *CHRM5* (173 bp), were amplified in most tissue cDNA samples (+) while the corresponding total RNA was used to control for genomic contamination (-). Nucleic acid size markers in base pairs are indicated.

Expression of *mAChR* mRNA in tree shrew ocular tissues was examined using RT–PCR. Figure 1 shows the amplification products for *CHRM1* (365 bp), *CHRM2* (197 bp), *CHRM3* (325 bp), *CHRM4* (394 bp), and *CHRM5* (173 bp) receptor genes in the different tissues investigated. The iris/ciliary body, retina, choroid, and sclera expressed all five muscarinic receptor subtypes while *CHRM2* expression was not detected in the cornea. Amplified products for all the receptor subtypes were also detected in samples isolated from cultured tree shrew scleral fibroblasts. An additional band in some of the *CHRM4* amplifications was due to primer-dimer formation as assessed by real-time PCR melt curves while there were no other contaminating products. For each *mAChR* subtype, amplifications were performed using the respective total RNA tissue sample as a template. Therefore, the amplifications were controlled for genomic contamination. No products were observed in any of these control amplifications. Purification and sequencing of the respective muscarinic receptor products showed a high identity to the published human receptor sequences (*CHRM1* 93%, *CHRM2* 83%, *CHRM3* 89%, *CHRM4* 93%, and *CHRM5* 82%).

**Figure 2:**
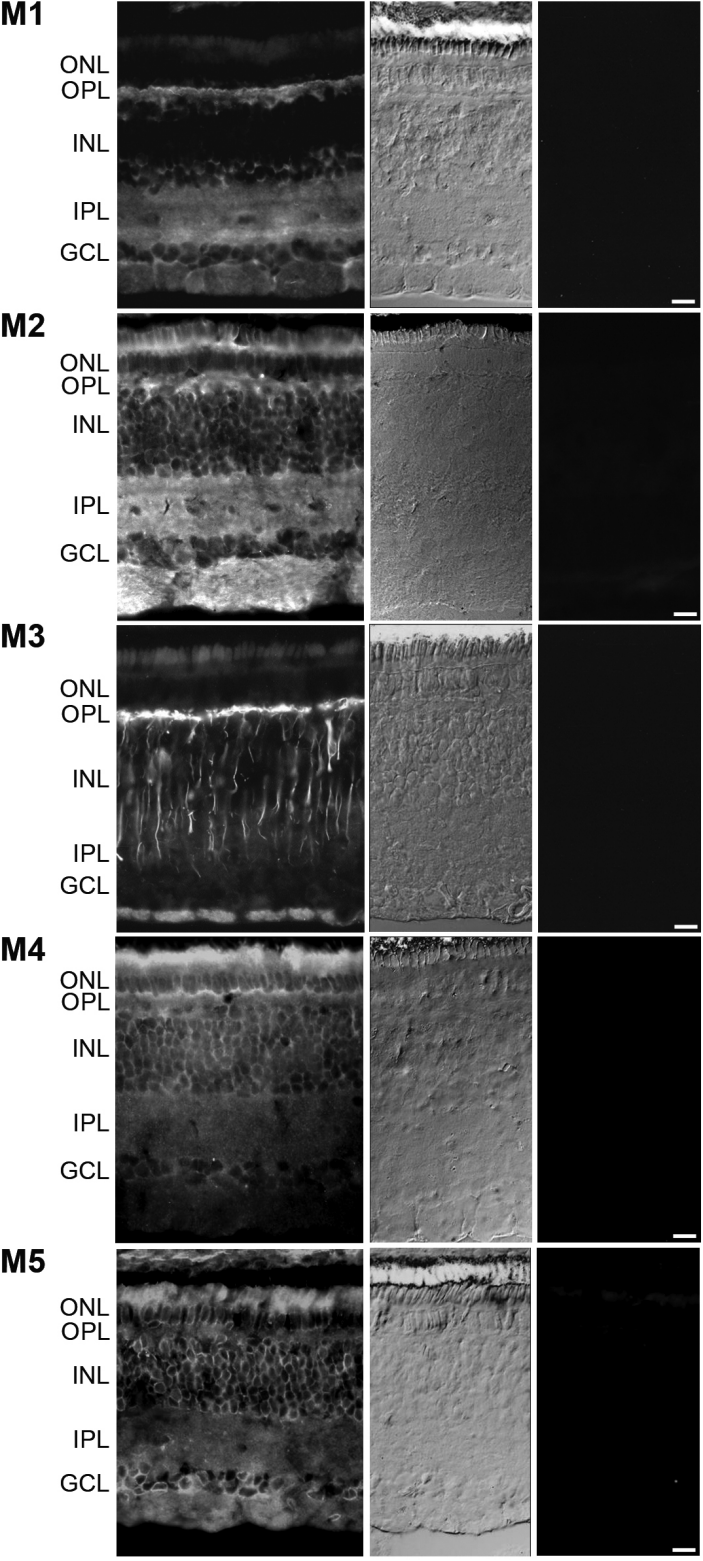
Retinal muscarinic receptor subtype protein expression in the tree shrew. Tree shrew posterior eye cups were fixed in 4% paraformaldehyde and reacted with muscarinic monoclonal antibodies to their respective receptor subtypes. A FITC-labeled secondary antibody was used to visualize the protein distribution in the retina (first column). The corresponding bright-field section (second column) and negative controls (last column) are also shown. The layers of the retina are highlighted, and the scale bar represents 20 μm. ONL, outer nuclear layer; OPL, outer plexiform layer; INL, inner nuclear layer; IPL, inner plexiform layer; GCL, ganglion cell layer; NFL, nerve fiber layer.

Localization of the M1-M5 mAChR proteins was investigated in the retina, choroid, and sclera using specific monoclonal antibodies directed against the respective receptor subtypes. The distribution and stratification of muscarinic receptors in the tree shrew retina are shown in Figure 2 (first column) together with the respective bright-field sections and negative controls (middle and last columns, respectively).

Relatively selective retinal expression was observed for the M1, M2, and M3 receptors. M1 immunoreactivity was localized to the outer (OPL) and inner (IPL) plexiform layers while the somata of cells in the inner nuclear layer (INL) and ganglion cell layer (GCL) exhibited limited staining. The M2 receptor was localized to the outer segments of the photoreceptor layer, OPL, IPL, and the nerve fiber layer (NFL) while the M3 receptor exhibited a quite distinctive expression in the OPL and NFL with processes extending through the INL and terminating at the IPL, which may be consistent with Müller cell expression. The specificity of the detection is evidenced by the blocking of the immunoreactivity after the addition of synthetic neutralizing peptides to the M1, M2, and M3 receptors (Figure 2, last column).

More diffuse retinal immunoreactivity was observed for the M4 and M5 receptors with stronger staining observed in the outer segments of photoreceptors and the OPL for M4 while M5 staining was strongest in the outer segments of photoreceptors and around the somata of cells in the INL and GCL. Again, the addition of the respective neutralizing peptides blocked all immunoreactivity.

**Figure 3:**
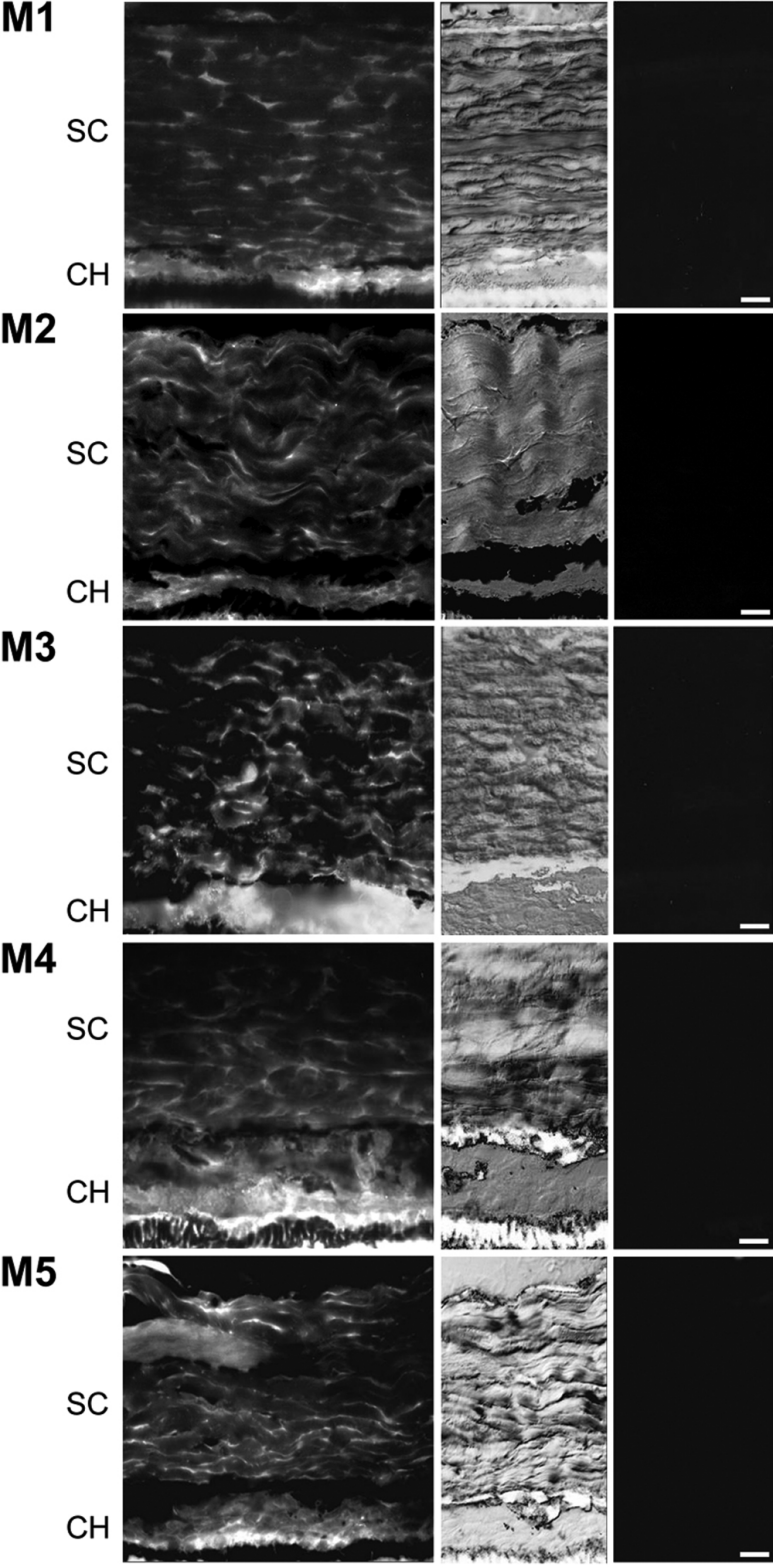
Choroidal and scleral muscarinic receptor subtype protein expression in the tree shrew. Tree shrew posterior eye cups were fixed in 4% paraformaldehyde and reacted with muscarinic monoclonal antibodies to the respective receptor subtypes. A FITC-labeled secondary antibody was used to visualize the protein distribution in the choroid (CH) and sclera (SC; first column). The corresponding bright-field section (second column) and negative controls (last column) are also shown. The choroid (CH) and sclera (SC) are labeled, and the scale bar represents 20 μm.

Figure 3 shows the distribution of the muscarinic receptor subtypes in the choroid and sclera. All receptors (M1-M5) showed staining in the choroid and sclera with scleral staining appearing between the collagen fiber bundles. All staining was blocked by the addition of the respective neutralizing peptides (Figure 3, last column).

As muscarinic receptor antagonists are able to inhibit myopia development both in animal models [28,29,33,34] and in humans [30,31] and there is conflicting data regarding the regulation of these receptors during myopia, real-time PCR was used to determine whether muscarinic receptor gene expression was altered in the tree shrew during experimental myopia. Each of the receptor subtypes was assessed at two time periods during myopia development, one representing a time before significant structural change (1 day) and the other after measurable ocular elongation (5 days). These time points were chosen to assess whether the *mAChR*s were actively involved in the early stages in myopic eye growth. Biometric data for the 5 day occluded group and age-matched normal group is shown in Table 2. In the 5 day occluded group, relative myopia had developed during the treatment period (treated eye–control eye, −8.1±0.6 D, p<0.0001). Most of the corresponding increase in eye size (treated–control eye, 0.20±0.2 mm, p<0.001) could be attributed to an increase in vitreous chamber depth (treated eye–control eye, 0.17±0.01 mm, p<0.0001) with anterior chamber depth and lens thickness not significantly altered (data not shown; p=0.46 and p=0.25, respectively). There was no difference between the normal age-matched animals and the control eyes from the 5 day occluded group.

**Table 2:** Ocular measures for normal tree shrews and those undergoing five days of monocular deprivation.

**Ocular measures**	**Normal group**	**Myopic group**		
	**Normal eyes**	**Control eyes**	**Treated eyes**	**Treated–control eyes**
Refraction (D)	8.6±0.1	8.4±0.1	0.3±0.5	−8.1±0.6**
Vitreous chamber depth (mm)	2.79±0.01	2.82±0.02	2.98±0.02	0.17±0.01**
Axial length (mm)	7.12±0.02	7.09±0.04	7.29±0.06	0.20±0.02*

Tree shrew ocular measures were taken before the isolation of the tissue samples. Refraction, vitreous chamber depth, and axial length are shown for the normal (visually unmanipulated) animals and the 5 day monocular deprived (myopic) group. Data are shown as mean±SEM, n=6. The asterisk indicates p<0.001, and the double asterisk indicates p<0.0001.

**Figure 4:**
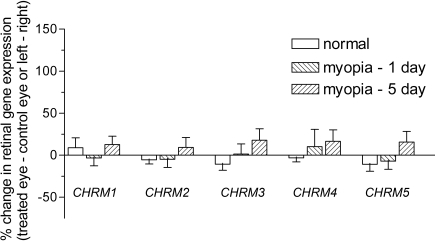
Regulation of retinal muscarinic receptor subtype gene expression during myopia development. The regulation of the five *mAChR*s was assessed in the tree shrew retina using real-time PCR and the DNA-binding dye, SYBR green I. Data was calculated relative to the housekeeping gene, *HPRT*, and expressed relative to the contralateral control eye (for myopic groups) or left/right eyes (for normal groups). Data are shown as the mean percentage change±SEM (n=6).

**Figure 5:**
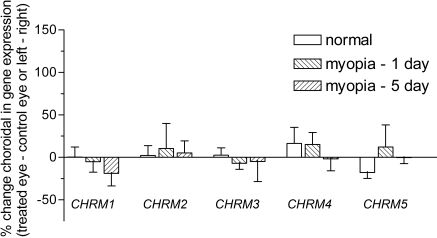
Regulation of choroidal muscarinic receptor subtype gene expression during myopia development. The regulation of the five *mAChR*s was assessed in the tree shrew choroid using real-time PCR and the DNA-binding dye, SYBR green I. Data was calculated relative to the housekeeping gene, *HPRT*, and expressed relative to the contralateral control eye (for myopic groups) or left/right eyes (for normal groups). Data are shown as the mean percentage change±SEM (n=5 for 1 and 5 day *CHRM2* gene data, all other data n=6).

**Figure 6:**
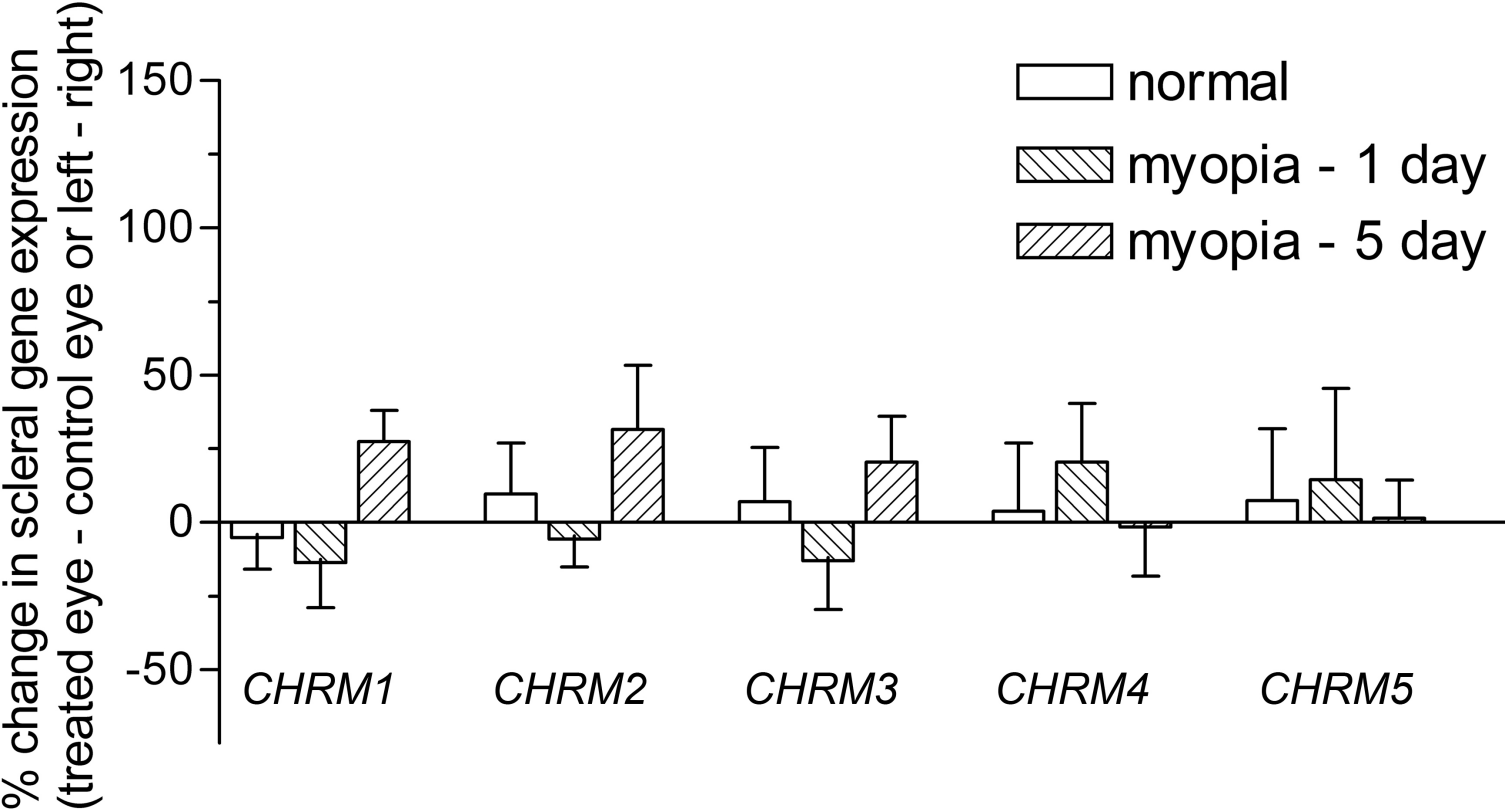
Regulation of scleral muscarinic receptor subtype gene expression during myopia development. The regulation of the five *mAChR*s was assessed in the tree shrew sclera using real-time PCR and the DNA-binding dye, SYBR green I. Data was calculated relative to the housekeeping gene, *HPRT*, and expressed relative to the contralateral control eye (for myopic groups) or left/right eyes (for normal groups). Data are shown as the mean percentage change±SEM (n=5 for 5 day myopia data, 1 day myopia and normal data, n=6).

Muscarinic gene expression was assessed in retinal, choroidal, and scleral tissues, each of which has been proposed as a potential site for atropine and/or pirenzepine action in halting myopia progression [48]. As observed in Figure 4, retinal muscarinic gene expression was not altered for any of the subtypes during the development of myopia (either 1 or 5 days) when treated (occluded) eyes were compared to their contralateral control (open) eyes. Data from the binocularly normal (visually unmanipulated) animals showed no difference in gene expression between left and right eyes. The choroidal (Figure 5) and scleral (Figure 6) tissue samples also showed no alteration in *mAChR* subtype gene expression during myopia development.

## Discussion

This study investigated mAChR gene and protein expression in the ocular tissues of the tree shrew and detailed subunit gene expression during the development of myopia. All five mAChR subtypes were found in the tree shrew iris/ciliary body, retina, choroid, and sclera while *CHRM2* mRNA was not detected in the cornea. *mAChR* subtype mRNA expression was not altered early in myopia development in the retina, choroid, or sclera either before or after significant axial elongation. Despite these tissues being implicated in the eye growth control, *mAChR*s do not play a direct role in the development of myopia in the tree shrew.

The tree shrew choroid and sclera contained the mRNA and protein for all five mAChR subtypes. Pharmacological and immunohistochemical localization techniques have been previously used to identify M1–M4 in the choroid [19,49-51]. However, this appears to be the first report of the M5 subtype. The scleral gene data reflects previous reports highlighting the expression of all *mAChR* subtype mRNA in the guinea pig and human sclera [38,40,52]. Immunohistochemical staining showed mAChR protein to be distributed evenly throughout the sclera. As there is limited innervation of the sclera and the labeling occurs between the collagen fiber bundles, it is likely that the mAChRs are localized to the scleral fibroblast processes. This was confirmed by the amplification of all five subtypes in cultured scleral fibroblasts. Similar receptor expression was found in human sclera and scleral fibroblast cells [38]. Chick scleral chondrocytes have also been suggested to express mAChRs due to their regulation via muscarinic antagonists [53]. The non-neuronal origin of scleral mAChR expression is not unusual, with other non-neural ocular tissues, such as the lens expressing mAChR subtypes [52]. Despite the constituents of the cholinergic system being present in several other cell systems, which are non-neuronal in origin [54], its functional role remains unclear.

The tree shrew iris/ciliary body complex also expressed all five mAChRs, which is in agreement with reports showing mRNA [14,15] and protein [14] expression for all five receptors in the human iris/ciliary body. Similarly, the mRNA and protein for all five mAChR subtypes was demonstrated in the tree shrew retina/RPE complex. This is consistent with reports that the mRNA for all five receptors has been found in the human retina [52]. Several other reports have also demonstrated the expression of individual subtypes such as M1, M2, M3, and M4 in retinae from various animals [13,23,55,56]. The distribution of the M1-M3 receptors in the plexiform layers of the retina is in agreement with previous localization of these subtypes [50,56,57]. However, while M4 expression in the photoreceptors, INL, and GCL is similar to that reported for chick retina [50], the receptor expression was generally more diffuse in the tree shrew. These observations may represent specific differences in the distribution of retinal mAChRs between the tree shrew and the chick or alternatively, differences in antibody specificities. To the best of our knowledge, this appears to be the first report showing M5 protein expression across the retina. While the current data shows its distribution to be similar to the M4 receptor, the lack of specific M5 antagonists at present makes its retinal role unclear. However, expression of M5 in the fish RPE may be involved in pigment granule dispersion [58].

The *CHRM1*, *CHRM3*, *CHRM4*, and *CHRM5* subtypes were identified in the cornea, which is supported by Lind and Cavanagh, [18] who detailed the expression of mAChR-like proteins of comparable molecular weights to the M3, M4, M5, and either the M2 or M1 receptors in cultured rabbit corneal cells. The *CHRM2* subtype mRNA was not detected in the current study depite the use of multiple amplification conditions. This subtype has been reported in bovine epithelial cells by Socci et al. [59,60] using the same technique and recently in human corneal epithelium and endothelium using immunohistochemistry. While this discrepancy may reflect species differences, it is difficult to prove that a protein is not expressed in a particular tissue. This is evidenced by the fact that the *CHRM1*, *CHRM3*, and *CHRM4* transcripts were not detected in the Socci et al. [59,60] study and the M1 and M3 receptors were not found in the human cornea.

Despite the presence of all receptor subtypes in the tree shrew retina, choroid, and sclera, *mAChR* subtype gene expression was not altered after 1 (before significant structural change) or 5 (after significant ocular elongation) days of myopia development. This is somewhat surprising if mAChR signaling is directly involved in eye growth since these tissues have been implicated in the local control of eye growth and the stimulation of mAChRs is known to alter subtype gene expression [36,61]. However, these data are generally consistent with previous work investigating the cholinergic system during myopia development. Within the retina, receptor density [27,39,62], levels of acetylcholine [63], and the activity of choline acetyltransferase [64] have all been reported to remain unchanged during the development of myopia. Likewise, studies on choroidal receptor density and affinity have shown no myopia-induced difference [39] despite earlier evidence of altered choline acetyltransferase activity in the choroid of form-deprived chicks [64].

Muscarinic receptors have long been implicated in myopia development due to the inhibitory effects of mAChR antagonists such as atropine, pirenzepine, and himbacine [26,29,34]. Yet, while the efficacy of these drugs is not a result of ocular toxicity [65], the mechanism remains unclear. Based on the retinal data showing unchanged receptor density/activity, the high doses required to inhibit experimental myopia, and the inability of quisqualic acid treatment to affect atropine-induced prevention of myopia [37], an extra-retinal site has been proposed. The current gene expression data supports a non-retinal site of action for these drugs. However, it provides no evidence for choroidal and/or scleral *mAChR* involvement during myopia development. While the investigation of choroidal and scleral mAChR protein expression would be required to discount post-translational alterations during myopia, an indirect role for mAChRs in eye growth appears more likely.

One such indirect effect of muscarinic antagonists on eye growth was proposed by Schwahn et al. [66]. In their study, an in vitro application of atropine induced a spreading depression in the retinal ERG response while the inhibition of myopia in vivo was accompanied by an increase in dopamine release. Such data lead to the hypothesis that atropine may interrupt the ocular growth signal via excessive retinal dopamine release. Whether such an effect would be via receptoral or non-receptoral mechanisms is unclear. However, the capacity of antagonists to utilize non-muscarinic mechanisms has been previously reported [67].

The current study directly contradicts the data of Lui et al. [40] where M1- and M4-specific increases in mAChR gene and protein expression were observed in the guinea pig during myopia development. This discrepancy may reflect species differences or methodological issues. While the guinea pig is becoming a more commonly used model in eye growth studies, there are limited data describing the scleral response (structural and biochemical) during myopia development. Therefore, it is difficult to compare the two models. An important consideration in comparing the two studies is that Liu et al. [40] induced myopia over a 21 day period rather than the 1 and 5 day period used in the current study. Thus, the observed alterations in M1 and M4 receptor expressions may reflect later changes arising from the enlarged eye rather than reflecting a causal relationship with eye growth. With respect to methodological issues, the guinea pig gene expression data was estimated from ethidium bromide band intensity using non-competitive end point PCR. Quantitative assessment of mRNA expression requires the measurement of the log-linear phase of the PCR amplification curve since the linear phase (end point) of the amplification curve can be affected by technical limitations such as primer-dimer formation and reduced reagent concentrations. Therefore, the linear phase provides limited quantitative information [68].

An earlier report of M1 and M4 regulation in the retina, choroid, and sclera after pirenzepine inhibition of myopia development in the guinea pig [69] could also be interpreted as demonstrating a direct muscarinic eye growth mechanism. Such an interpretation could be supported by data showing pirenzepine, being M1 selective, and himbacine (M4 selective) specifically inhibit experimentally induced myopia. However, an addition of muscarinic antagonists is known to result in increased receptor expression [61], and it is unclear from the report whether the pirenzepine-induced increase in the M1 and M4 receptors directly reduced the myopia or was merely a result of it. Nevertheless, while there appears to be limited if any change in the ligands or receptors within the acetylcholine system in most animal models of myopia development, it is possible that alterations in downstream muscarinic signaling may directly modulate eye growth. For example, mAChR internalization, desensitization, and downregulation of signal transduction elements can also function to modulate receptor-mediated function [61]. Further work will be required to investigate these possibilities.

Tree shrew retina, choroid, and sclera express all five mAChR subtypes, but there is no alteration in tissue expression levels during myopia development. The lack of any regulatory change in the gene expression of the retinal, choroidal, and scleral receptors suggests an indirect role for the acetylcholine system in eye growth. Future work will target alternate regulatory pathways outside the acetylcholine system to characterize the mechanism used by muscarinic antagonists to inhibit myopia development.
